# The effect of Riociguat on cardiovascular function and efficiency in healthy, juvenile pigs

**DOI:** 10.14814/phy2.14562

**Published:** 2020-09-12

**Authors:** Torvind Næsheim, Ole‐Jakob How, Truls Myrmel

**Affiliations:** ^1^ Cardiovascular Research Group Department of Clinical Medicine UiT The Arctic University of Norway Tromsø Norway; ^2^ Department of Anesthesiology University Hospital North Norway Tromsø Norway; ^3^ Department of Medical Biology Faculty of Health Sciences UiT The Arctic University of Norway Tromsø Norway; ^4^ Department of Cardiothoracic and Vascular Surgery University Hospital North Norway Tromsø Norway

## Abstract

**Introduction:**

Riociguat is a soluble guanylate cyclase stimulator approved for the treatment of pulmonary hypertension. Its effect on cardiometabolic efficiency is unknown. A potential cardiac energy sparing effect of this drug could imply a positive prognostic effect, particularly in patients with right heart failure from pulmonary hypertension.

**Method:**

We infused Riociguat in six healthy juvenile pigs and measured the integrated cardiovascular effect and myocardial oxygen consumption. To assess the interplay with NO‐blockade on cardiac function and efficiency we also administered the NO‐blocker L‐ NAME to the animals after Riociguat.

**Results and Discussion:**

Infusion of 100 µg/kg Riociguat gave modest systemic vasodilatation seen as a drop in coronary and systemic vascular resistance of 36% and 26%, respectively. Right and left ventriculoarterial coupling index (Ees/Ea), stroke work efficiency (SWeff), and the relationship between left ventricular myocardial oxygen consumption (MVO_2_) and total mechanical work (pressure–volume area; PVA) were unaffected by Riociguat. In contrast, systemic and pulmonary vasoconstriction induced by L‐NAME (15 mg/kg) shifted the Ees/Ea ratio toward reduced SWeff in both systemic and pulmonary circulation. However, there was no surplus oxygen consumption, that was measured by the MVO_2_/PVA relationship after L‐NAME in Riociguat‐treated pigs. This suggests that Riociguat can reduce the NO‐related cardiometabolic inefficiency previously observed by blocking the NO pathway.

## Graphical abstract

The sGC stimulator Riociguat hamper the energy wasting effect of NO‐blocade in Health juvenile pig hearts

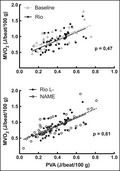

## 1 INTRODUCTION

Myocardial oxygen consumption (MVO_2_) is influenced by substrate metabolism, heart rate, wall tension and contractility (Braunwald, 1969). Under normal physiological conditions, these parameters are determined by the hormonal state of the individual. Importantly, adrenergic hormones and drugs increase contractility and relative oxygen consumption (Müller, How, & Jakobsen, 2010; Vasu et al., 1978). Previous studies in large animal models indicate that an elevated NO‐tone will improve myocardial efficiency, i.e., reduces MVO_2_ related to mechanical work (Suto et al., 1998). Riociguat, a soluble guanylate cyclase (sGC) stimulator, exhibits a vasodilatory and NO‐like effect by direct stimulation of the sGC enzyme, thus circumventing the need for NO stimulation and compensating for decreased NO sensitivity in vascular pathology. Riociguat could therefore have a favorable energetic profile equal to direct NO effects in the myocardium. This would be particularly beneficial in patients with pulmonary hypertension and right heart failure treated with Riociguat (Adempas®). To assess this possibility, we used a well‐established metabolic and energetic effect model (Korvald, Elvenes, & Myrmel, 2017) to determine the myocardial oxygen consumption and function during Riociguat infusion in healthy pigs. Studies have shown that there is a basal oxygen‐sparing level of NO in the myocardium (Loke, McConnell, & Tuzman, 1999; Recchia et al., 1999), and blocking this NO‐tone by infusion of L‐NAME has been shown to induce surplus MVO_2_ (Nordhaug, Steensrud, Aghajani, Korvald, & Myrmel, 2004). We hypothesized that Riociguat added to normal hearts would reduce MVO_2_ relative to mechanical work in the left ventricle and that the NO‐blocker L‐NAME would reverse this oxygen‐sparing effect.

## 2 METHODS

### 2.1 Animals

Six castrated male domestic pigs weighing 30 ± 5 kg were adapted to the animal department for 5–7 days. The pigs were fasted overnight before experiments, with free access to water. The experimental protocol was approved by the Norwegian Animal Research Authority according to the FDF reference; 2012/55972.

### 2.2 Surgical preparation

The pigs were premedicated with an intramuscular injection of 20 mg/kg ketamine (Pfizer AS, Oslo, Norway) and 1 mg of atropine (Nycomed Pharma, Oslo, Norway). Anesthesia was induced by intravenous injection of 10 mg/kg pentobarbital sodium (Abbott, Stockholm, Sweden) and 0.01 mg/kg fentanyl (Hameln Pharmaceuticals, Hameln, Germany). The animals were ventilated after intubation. Normal ventilation was defined as an PaCO_2_ of 40 ± 2 mmHg. A central venous catheter was placed through the left internal jugular vein and anesthesia was maintained throughout the experiment by a continuous infusion of 4 mg·kg^−1^·hr^−1^ pentobarbitone sodium, 0.02 mg·kg^−1^·hr^−1^ fentanyl, and 0.3 mg·kg^−1^·hr^−1^ midazolam (B. Braun, Melsungen, Germany). The circulating volume was maintained by a 20 ml·kg^‐1^·hr^−1^ continuous infusion of 0.9% NaCl supplemented with 1.25 g·L^−1^ glucose. Following sternotomy, the pericardium was removed, and the coronary arteries and pulmonary trunk were dissected free from connective tissue. The hemiazygos vein was then ligated.

### 2.3 Instrumentation

**Figure 1:**
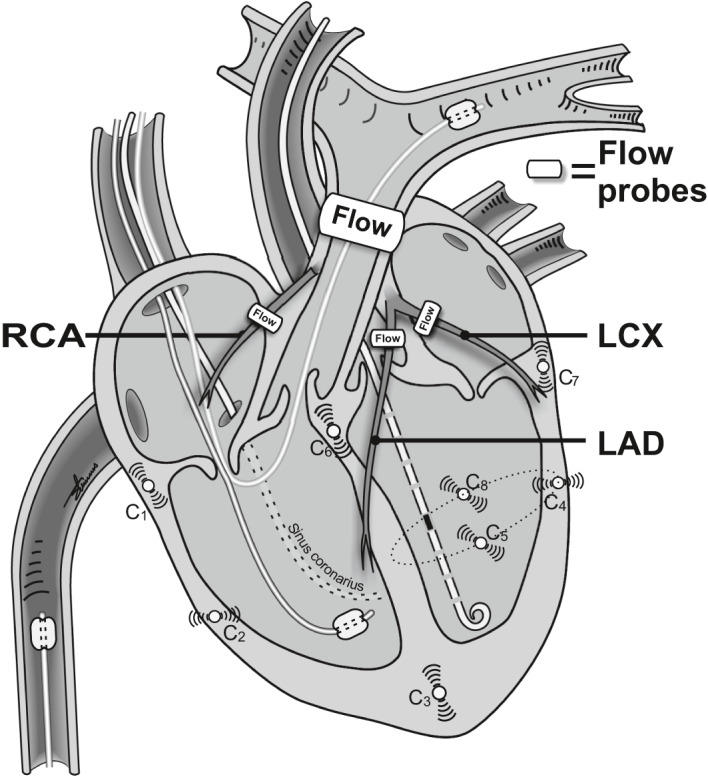
Schematic drawing of the sonometric crystal positions used for assessing right and left ventricular volumes (see equation 1 and 2). The placements of the crystals are in the subendocardial position. Crystal no. 8 is placed in the posterior wall of the left ventricle adjacent to crystal no. 5 in the anterior wall. “Flow” indicates time transit flow probes, RCA is the right coronary artery, LCX is the left circumflex coronary artery, LAD is the left anterior descending artery, C1 to C8 are sonometric crystals placed in the subendocardium. Balloon catheters are drawn in the right ventricle and pulmonary artery

A 7‐Fr pressure catheter (Millar MPVS Ultra, Houston, TX, USA) was inserted through an introducer sheath via the carotid artery into the left ventricle. A 5 Fr Swan‐Ganz catheter (Edwards Lifescience Corp. Irvine, USA) was advanced into the pulmonary artery. A 5 fr balloon catheter was floated from the superior caval vein into the right ventricle for pressure measurements. Central venous pressure was measured in the right atrium. The systemic arterial pressure was assessed from a vascular catheter in the abdominal aorta. An 8 Fr balloon catheter was introduced into the inferior caval vein and positioned just below the right atrium for intermittent preload reduction. A 4 Fr catheter with side holes was placed in the coronary sinus for the collection of coronary venous blood. Transit time flow probes (Transonic Systems Inc, Ithaca, NY, USA) were placed around the pulmonary trunk and the three coronary arteries. Eight sonometric crystals (Sonometrics Corporation, Ontario, Canada) were implanted subendocardially, as described in Figure 1. At the end of the experiment, the heart was stopped by injection of 20 mmol KCl (1 mmol/ml). The heart was then excised, and through a balloon catheter in the left main coronary artery, Evans Blue (Sigma‐Aldrich, Saint‐Louis, Missouri, USA) was injected, and the stained and unstained heart muscle was weighed.

### 2.4 Experimental protocol and drugs

The Riociguat dose was based on a dose‐response study in four animals (Næsheim, How, & Myrmel, 2020), targeting a mean systemic blood pressure at 50 mmHg. In this study, the experiments were conducted using a repeated measures design. The ganglion blocker Hexamethonium chloride (Sigma‐Aldrich Missouri, USA) 15 mg/kg was given to avoid autonomous reflexes during interventions and measurements (Douglas, 1952). Heparin, 2,500 IU, was given intravenously after instrumentation to prevent clotting of catheters. Following surgery, the pigs were allowed to rest for 30 min before baseline measurements.

Riociguat (100 µg/kg) was then given as an intravenous bolus, and the second recording was carried out after another 30 min. Finally, L‐NAME (15 mg/kg) was given, also as a bolus. The dose of L‐NAME was based on an earlier dose‐response study in our lab (Nordhaug et al., 2004). The last recording was carried out after the hemodynamic parameters had stabilized after approximately 15 min. At each time point, a full set of cardiac function parameters and vascular measurements were recorded. Assessment of cardiometabolic efficiency was done according to an established protocol (Korvald et al., 2017) with some modification particularly related to measurements of cardiac volumes by sonometric crystals (Feneley et al., 1990). A downloading protocol was carried out after every drug intervention: By stepwise inflating the balloon catheter in the caval vein, stroke work, coronary flow, and arterial–to‐coronary venous oxygen difference was recorded at 6–8 different preloads.

Riociguat was obtained from Chemoki Synthesi‐TECH, Jiangsu, China, as a dry powder. pH neutral solutions were prepared with DMSO (dimethyl sulfoxide) and a 1:1 solution of Transcutol, Diethylene glycol ethyl ether (Sigma‐Aldrich, Missouri, USA) and Cremophor, macrogolglycerol ricinolate (Sigma‐Aldrich Missouri, USA). We used 5% Transcutol and Chremophore solutions, and the volume ratio between DMSO, Transcutol, and Chremophore was 0.05:2.5:2.5. This solution was then further diluted with 0.9 mg・ml^‐1^ NaCl to a final concentration of test drug of 0.01 to 0.1 mg・mL^‐1^ depending on the dose to be given. The maximum DMSO concentration was 0.02%. L‐NAME, N‐omega‐nitro‐L‐arginine methyl ester, 15 mg/kg, was used as a nitric oxide synthetase inhibitor (Rees, Palmer, Schulz, Hodson, & Moncada, 1990).

### 2.5 Registration of data and analysis

Data were sampled, digitized and analyzed (using ADI MPVS Ultra and Powerlab hardware and LabChart Pro software v 8.1.8, Dunedin, New Zealand). Cardiac dimension data, obtained by the sonometric crystals (Figure 1), were calibrated to cardiac volumes at baseline with the use of intrathoracic Echocardiography (Philips IE33, Philips Healthcare). Echocardiography was also used to confirm the sphericity of the left ventricle at all time points. For all the following recordings, cardiac volumes were calculated from the sonometric data using the following formulas:

The volume of the left ventricle was calculated by the biplane area‐length formula as:

$$V_{\mathrm{lv}}=\frac{\pi }{6}D_{5‐8}^{2}\cdot D_{7‐3}$$

The volume of the right ventricle was estimated by a simplified shell subtraction model as:

$$V_{\mathrm{rv}}=\frac{\pi }{6}D_{6‐3}\left(D_{4‐2}^{2}‐D_{5‐8}^{2}\right)$$

D_n‐n_ indicates the cardiac dimensions between selected crystals, as shown in Figure 1. This ignores the volume of the intraventricular septum, and will therefore predictably overestimate right ventricular volumes. For assessment of end‐systolic elastance (Ees, maximal ventricular tension as an index of contractility) and V_0_ (unstressed ventricular volume), an abrupt reduction in preload was carried out by rapid inflation of the balloon catheter situated in the caval vein. Example loops from the left and right ventricle are presented in Figure 4. V_0_ was calculated at baseline for each pig and used as a constant for the remainder of the experiment. Arterial elastance, Ea, in both ventricles was calculated as.

Ea=ESP/SV

ESP is the end‐systolic pressure, and SV is the stroke volume (Morimont, Lambermont, & Ghuysen, 2008).

Stroke work efficiency, the ratio of total ventricular mechanical work delivered to the circulation, was calculated by stroke work (SW)/ pressure–volume area (PVA) (Vanderpool et al., 2015). Stroke work was calculated as:

$$\text{SW}=\left(P_{\max}‐\text{EDP}\right)\times \text{SV}$$

P_max_ is the maximum pressure in the ventricle (mmHg), EDP is the end‐diastolic pressure in the ventricle (mmHg), and SV is the stroke volume (ml). Pressure–volume area (PVA) was calculated as SW + potential energy (PE):

$$\text{PE}=\left(\text{ESP}\times \left(\text{ESV}‐V_{0}\right)\right)/2‐\left(\text{EDP}\times \left(\text{EDV}‐V_{0}\right)\right)/4)$$

ESV is the end‐systolic volume (ml), EDV is the end‐diastolic volume (ml), and V_0_ is the x‐axis intercept of the regression line of the end‐systolic pressure–volume relation at baseline.

Left ventricular mechanoenergetic efficiency was assessed by the linear regression of PVA and myocardial oxygen consumption (MVO_2_) at various workloads, where MVO_2_ was calculated as:

$${\text{MVO}}_{2}=\left(\text{Hb}\times {\text{avdO}}_{2}\times \text{LVCBF}\times 1.39\right)/\left(\text{HR}\times 20.2\right)$$

Hb is hemoglobin concentration (g/dl), avdO_2_ is the difference in arterial and great cardiac vein oxygen saturation (%), LVCBF is the left ventricular coronary blood flow (ml/min) or blood flow through the circumflex and left anterior descending arteries divided by left ventricular weight (LVW in gram (g), as described (Aghajani et al., 2004; Yao, Xue, Yu, Ni, & Chen, 2018)). HR is heart rate (beats per minute), 1.39 is a constant (ml O_2_/g Hb), and 20.2 is a factor for J/mL O_2_ to convert MVO_2_ to mechanical energy equivalents. In the MVO_2_/PVA framework the y‐axis intercept represents myocardial oxygen cost for excitation–contraction coupling and basal metabolism, while 1/slope represents cardiac efficiency of contractile work (Myrmel & Korvald, 2000).

### 2.6 Statistical analysis

**Figure 2:**
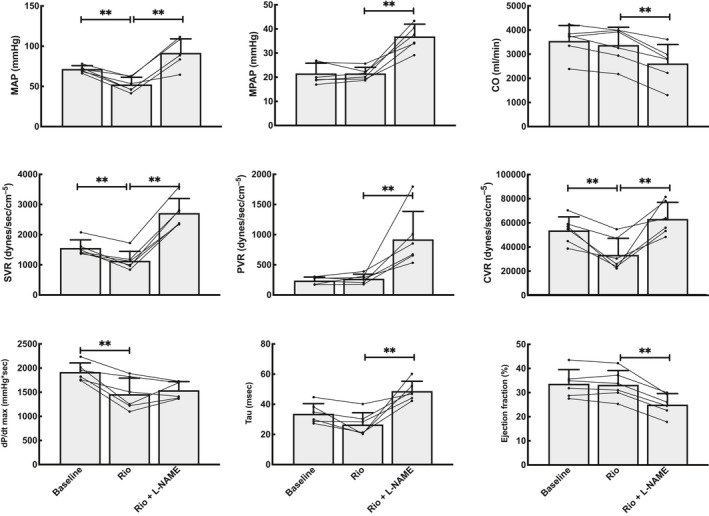
General hemodynamic measurements at baseline (Baseline), after a bolus of 100 µg/kg Riociguat (Rio) and finally after adding 15 mg/kg L‐NAME. MAP is the mean systemic arterial pressure, MPAP is the mean pulmonary arterial pressure, CO is the cardiac output. SVR, PVR, and CVR are the systemic, pulmonary and coronary arterial resistances. dP/dT_max_ is the maximum slope of left ventricular pressure development. Tau is the time constant of isovolumetric relaxation. EF is the left ventricular ejection fraction. *N* = 6, ** denotes *p* < .01 compared to the previous phase of the experiment

**Figure 3:**
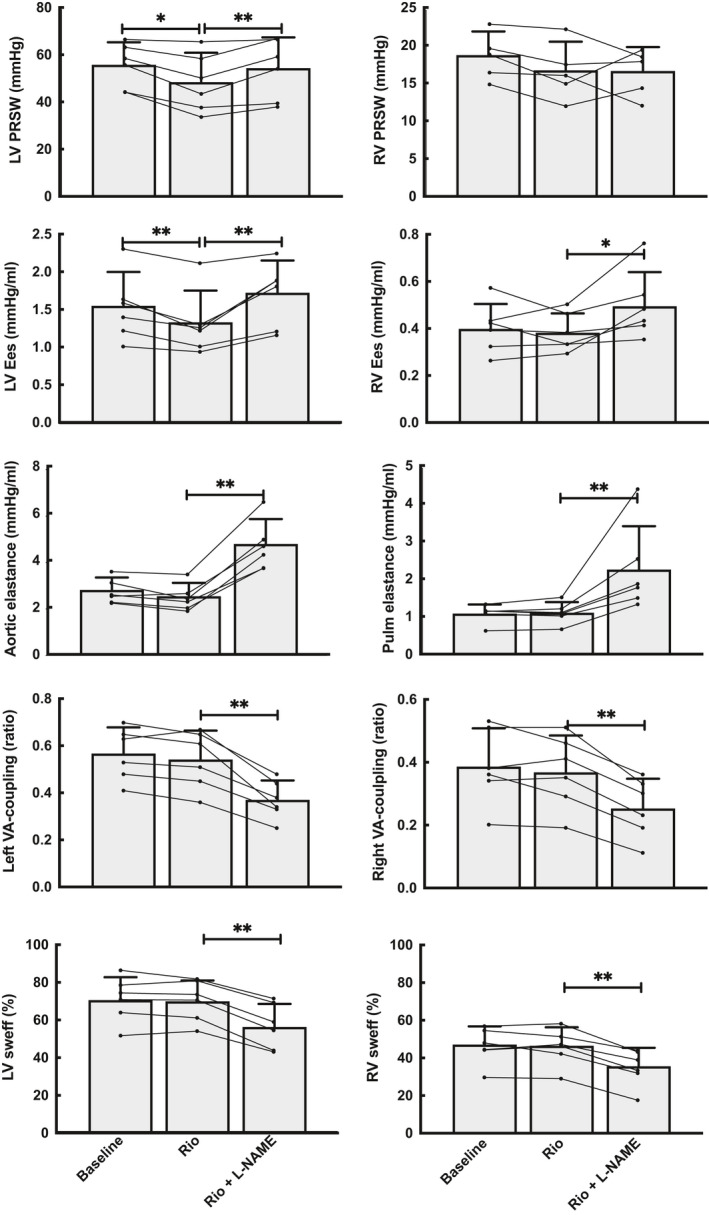
Ventriculoarterial coupling (VA coupling) and stroke work efficiency (SWeff) in both the left (LV) and right (RV) ventricles after subsequent boluses of Riociguat and L‐NAME (see Figure 2). Left column displays data from the systemic circulation and right column from the pulmonary vasculature. VA coupling was calculated as the ratio of ventricular end‐systolic and arterial elastance (Ees/Ea). SWeff is the portion of total ventricular mechanical work (pressure–volume area, PVA) measured as external pressure and volume work (SW). *N* = 6, * *p* < .05, ** *p* < .01 compared to the previous measuring point in the experiment

Calculations and statistical analyses were performed using a spreadsheet (Microsoft Excel 365, Microsoft, USA) and a statistical package (IBM SPSS Statistics for Windows, IBM Corp, Version 25.0. Armonk, NY). Values are presented as the mean ± standard deviation. Mixed model analysis with pig identity as random effect, with allowance for variation in both intercept and slopes between pigs, was performed on the graph in Figures 2 and 3. Pairwise comparisons with least significant difference were evaluated between time points. The autoregressive covariance structure was modeled to give the best fit of data. Mixed model linear regression with pig identity as random effect was used on cardiac energetics data. For these calculations, MVO_2_ and the product of MVO_2_ and the dummy variable for medication were used as fixed effects (linear regression Figure 7). *p*‐values **<.01 * <.05 were regarded as statistically significant.

## 3 RESULTS

### 3.1 Dose‐response‐study

Increasing doses of Riociguat up to 100 µg/kg induced progressive systemic vasodilatation and hypotension. No significant effects were seen in the pulmonary vasculature. At the maximum dose tested the mean systemic pressure reached the predefined threshold of 50 mmHg (manuscript in press). Heart rates in the control state during Riociguat infusion and after L‐NAME bolus were 112 ± 9 min^‐1^, 111 ± 14 min^‐1^ and 111 ± 11 min^‐1,^ respectively, with no significant difference (Table 1).

**Table 1:** Dose‐response study of Riociguat

Riociguat, *n* = 4					
	Vehicle	10 µg/kg	20 µg/kg	50 µg/kg	100 µg/kg
MAP, mmHg	88 ± 21	87 ± 22	66 ± 11^#^	56 ± 13^#^	50 ± 9^#^
MPAP, mmHg	25 ± 23	22 ± 2	23 ± 5	25 ± 7	25 ± 8
SVR, dynes/s/cm^−5^	1,066 ± 248	957 ± 204	760 ± 180^#^	568 ± 124^#^	497 ± 161^#^
PVR, dynes/s/cm^−5^	166 ± 109	146 ± 87	162 ± 76	179 ± 76	167 ± 65

Note

Dose‐response data for Riociguat submitted in other publication. MAP, Mean systemic arterial pressure; MPAP, Mean pulmonary arterial pressure; SVR, Systemic vascular resistance; PVR, Pulmonary vascular resistance. Values are mean ± standard deviation. Significance levels between doses of test drug against baseline.

^#^ =*p* < 0.05 (mixed model statistics with pig identity as random effect).

### 3.2 Effects on vascular tone

A bolus of 100 µg/kg Riociguat gave modest systemic vasodilatation, including the coronary circulation, as seen by a drop in coronary and systemic vascular resistance of 36% ± 21% (*p* = .01) and 26% ± 18% (*p* = .00), respectively (Figure 2). No significant effect was seen in the pulmonary vasculature.

A bolus of the NOS inhibitor L‐NAME (15 mg/kg) after Riociguat resulted in both pulmonary and systemic vasoconstriction as seen by a 2.5 ± 1.6 (*p* = .01) and 1.5 ± 0.2 (*p* = .00) fold increase in PVR and SVR, respectively.

### 3.3 Cardiac effects

The bolus of Riociguat did not alter cardiac output or ejection fraction (Figure 2). Left ventricular maximum pressure development (LV dP/dt) decreased by 24% ± 12% (*p* = .00), while no change in this index was seen for the right ventricle. Stroke work was reduced by 20% ± 8% (*p* = .00) for the left, but not the right ventricle. Left ventricular end‐systolic pressure–volume relation, i.e., elastance (LV E_es_), was reduced by 14% ± 14% (*p* = .01). Right ventricular elastance (RV E_es_), remained stable. Preload recruitable stroke work (PRSW) decreased by 13% ± 7% (*p* = .01) for the left ventricle but did not change for the right ventricle. The diastolic relaxation index, Tau, was unaltered for the left ventricle. Ventricular dimensions remained unchanged.

L‐NAME decreased cardiac output by 23% ± 7% (*p* = .00) and left ventricular ejection fraction (EF) decreased from 33% ± 5% to 25% ± 4% (*p* = .00). Heart rate was unchanged. Reduction in EF was accompanied by an increase in end‐systolic volumes for both ventricles; 11% ± 8% (*p* = .010) for the left ventricle and 12% ± 10% (*p* = .04) for the right ventricle. Left ventricular elastance increased by 29% ± 23% (*p* = .01) and right ventricular elastance increased by 18% ± 10% (*p* = .02) (Figures 5 and 6). Left ventricular PRSW increased by 12% ± 8% (*p* = .00), while no effect was found on right ventricular PRSW.

### 3.4 Effects on ventriculoarterial coupling

Riociguat did not impact the relationship between contractility and afterload demonstrated by an unchanged ventriculoarterial coupling and stroke work efficiency for both the systemic and pulmonary circulation. L‐NAME, on the other hand, elevated afterload proportionately more than contractility, causing an offset in the Ees/Ea relationship for both ventricles. This resulted in impaired stroke work efficiency in both left and right ventricle by 19% ± 5% (*p* = .00) and 25% ± 10% (*p* = .00) (Figures 3, 5 and 6).

**Figure 4:**
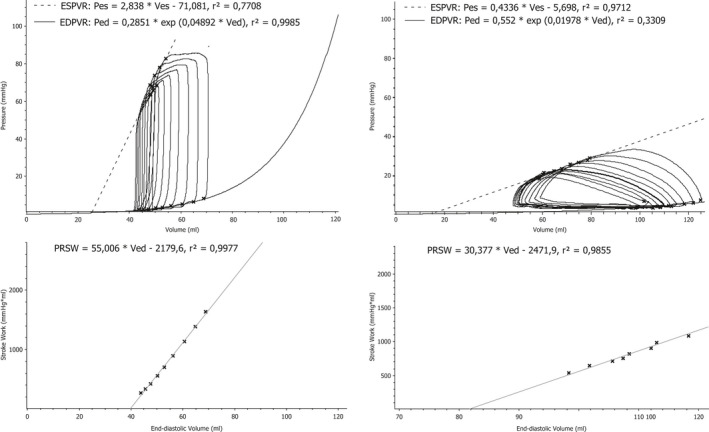
Screenshot of actual recordings from one pig. Pressure–volume loops and calculated preload recruitable stroke work (PRSW) during reduction in preload by inflation of the Fogarty balloon catheter in the inferior caval vein. Left panels are from the left ventricle. Right panels are from the right ventricle. Top panels are the pressure–volume loops with linear curve fitting to the maximum pressure/volume relationship (ESPVR) and curvilinear curve fitting of the end‐diastolic pressure/volume relationship (EDPVR). The lower panels show the preload recruitable stroke work for the left and right ventricles, respectively

**Figure 5:**
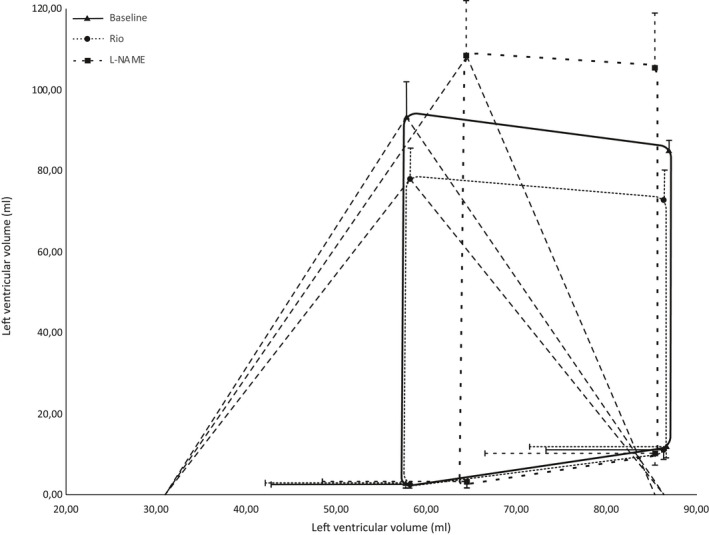
Ventriculoarterial coupling, left ventricle. Schematic pressure–volume curves for the left ventricle based on the average values of the end diastolic pressure–volume relationship (EDPVR), the pressure–volume relationship after isovolumetric contraction, the end systolic pressure–volume relationship, and the pressure–volume relationship at the start of diastole. Curves are fitted through these four points. Also shown is the average volume at the x‐axis intercept of the ESPVR line at baseline, V0. Left ventricular elastance is the slope of the ESPVR line (Ees). The slope from the end systolic pressure–volume point to the X‐axis intercept of the end‐diastolic volume is the aortic elastance (Ea). Error bars are standard deviation for pressures and volumes. *N* = 6

**Figure 6:**
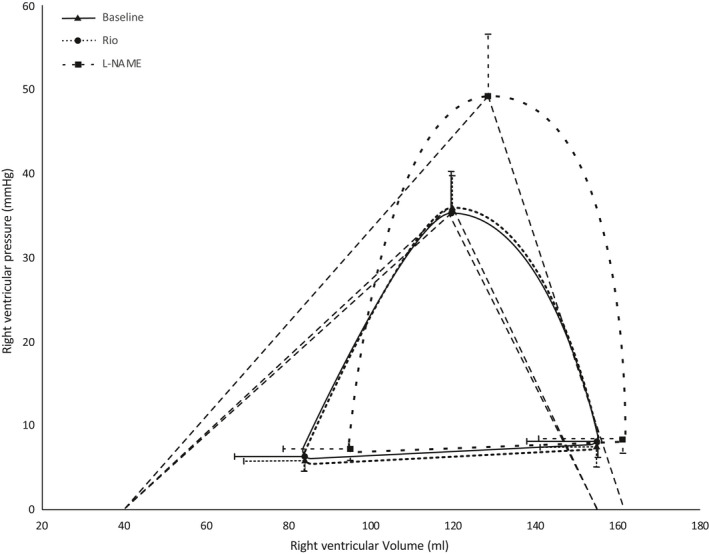
Ventriculoarterial coupling, right ventricle. Schematic pressure–volume curves for the right ventricle based on the average values of the end diastolic pressure–volume relationship (EDPVR), the maximum systolic pressure–volume relationship, and the pressure–volume relationship at the start of diastole. Curves are fitted through these three points. Also shown is the average volume at the x‐axis intercept of the end systolic pressure–volume line (ESPVR line) at baseline, V0. The slope of the ESPVR line is the right ventricular elastance (Ees) and the slope from the maximum systolic pressure–volume point to the X‐axis intercept of the end diastolic volume is the pulmonary arterial elastance (Ea). Error bars are standard deviation for pressures and volumes. *N* = 6

Figure 4 shows an example of pressure–volume relations during preload reductions in one pig at baseline, and Figures 5 and 6 the detailed effects of Riociguat and L‐NAME on the time‐varying interaction of the two ventricles with their afterloads (ventriculoarterial coupling). These compilations demonstrate that Riociguat mainly acts by reducing the afterload on the left ventricle giving reduced stroke work due to lesser pressure work but no altered ventricular volumes. L‐NAME increases the arterial elastance, reduces the volume‐related stroke work with an increased end‐systolic volume (reduced ejection fraction) with a resulting reduced cardiac output.

### 3.5 Effects on mechanoenergetic efficiency

**Figure 7:**
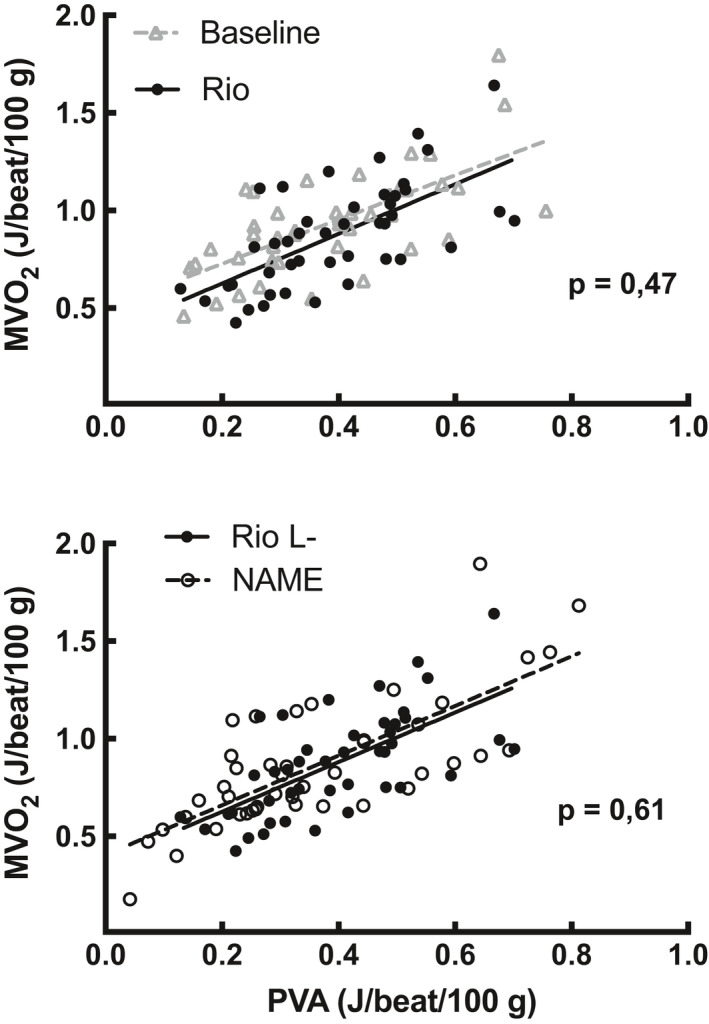
Mechanoenergetic efficiency of the left ventricle. Mechanoenergetic efficiency was calculated as the pressure–volume area (PVA) related to myocardial oxygen consumption (MVO_2_) at a range of workloads. The panels display the pooled scatter and linear regression of MVO_2_/PVA recordings from six pigs

The mechanoenergetic efficiency of the left ventricle was unaffected by Riociguat, displayed by unaltered MVO_2_/PVA relationship with regard to both slope and intercept. Blocking NO by applying a subsequent L‐NAME bolus did not impact the relative oxygen consumption in Riociguat‐treated hearts (Figure 7).

## 4 DISCUSSION

The main finding in this study was an observed neutral effect of Riociguat on myocardial oxygen consumption. There was no shift in the MVO_2_/PVA relationship for Riociguat, indicating that stimulation of sGC did not influence myocardial metabolism in healthy pigs in the dose given in this study. The dose chosen was the highest dose possible to administer while avoiding excessive hypotension. Of notice, the subsequent administration of the NO‐blocker L‐NAME did not increase the relative oxygen consumption in the myocardium, and this suggests that the expected surplus MVO_2_ after using L‐NAME (Nordhaug et al., 2004) was attenuated by this dose of Riociguat.

The indication for using Riociguat is idiopathic pulmonary artery hypertension (PAH) and chronic thromboembolic pulmonary hypertension (CTEPH), two out of five categories of pulmonary hypertension (PH), according to the World Health Organization (Farber, Miller, & Poms, 2015). Inadequate NO‐mediated vasodilation is believed to have an essential role in the pathogenesis of PAH. Thus, stimulating the NO‐sGC‐cGMP‐PKG pathway is a seemingly attractive treatment target (Ghofrani, Humbert, & Langleben, 2017). Indeed, inhibition of the cGMP degradation by the PDE5 inhibitor Sildenafil has improved outcomes for patients with PAH (Galiè et al., 2005). However, PAH is associated with reduced NO availability and reduced sensitivity of the sGC as caused by oxidative stress that may restrict the effectiveness of PDE5 inhibitors (Hoeper, Simonneau, & Corris, 2017; Lang, Kojonazarov, & Tian, 2012). Riociguat works through the same pathway; by stimulating sGC partly independent of available NO and with higher potency than NO when sGC has undergone oxidation in pathological tissues (Thoonen et al., 2015). While efforts have been taken to investigate the vascular effects of Riociguat in the diseased state with particular emphasis on PAH, little attention has been on the physiological effects of the drug on the heart in general and the right ventricle in particular. This is relevant since PAH is the leading cause of right heart failure, and heart failure determines the prognosis in PAH (Kylhammar, Kjellström, & Hjalmarsson, 2018; Vonk‐Noordegraaf et al., 2013).

The impact of NO on contractility and cardiometabolic efficiency has been an area of particular attention. NO and cGMP seem to influence contractile function in a concentration‐dependent bidirectional fashion. In an in vitro model, the effect of NO on cardiomyocytes was found to be dependent on intact endothelium. In this study, cyclic GMP affected the contractile response of the myocytes via a biphasic inhibition and then stimulation of cGMP‐dependent cAMP phosphodiesterase inhibitor through modulation of intracellular Ca^2+^ levels and myofilament Ca^2+^ sensitivity. Increasing NO and cGMP levels first induced an increase in contractility followed by a fall at high concentrations(Mohan, Brutsaert, Paulus, & Sys, 1996). In a human study on cardiac failure, on the other hand, blockade of NO synthetase increased inotropic response to β‐adrenergic stimulus (Hare, Givertz, Creager, & Colucci, 1998). The complex interaction between the NO‐sGC‐cGMP‐pathway and contractility makes it challenging to predict the exact optimum dose of NO‐donors or sGC activators or stimulators when aiming for therapeutically altered contractility.

In our study using sympathetically blocked healthy animals, Riociguat had a predominantly systemic vasodilatory effect. The concomitantly observed hemodynamic effects of the drug can, for a large part, be a consequence of this unloading affect. The effects on contractile function (elastance and PRSW), ventriculoarterial matching (Ees/Ea ratio) and left ventricular pump efficiency (SW efficiency) were overall minor. The model is therefore well designed to pick up relatively minor direct metabolic or oxygen‐consuming effects of test‐drugs. Using the same experimental setup, in earlier studies, we have shown that NO‐blockade, inotropic drugs and systemic infections have clear metabolic effects in the myocardium evaluated in the frame of an MVO_2_/PVA analysis (Aghajani, Korvald, Nordhaug, Revhaug, & Myrmel, 2004; Aghajani, Nordhaug, et al., 2004; Elvenes et al., 2000; How et al., 2005; Korvald, Elvenes, Ytrebø, Sørlie, & Myrmel, 1999; Nordhaug, Steensrud, Korvald, Aghajani, & Myrmel, 2002).

There is consistent evidence that blocking enzymes in the NO pathway causes reduced cardiac efficiency by surplus myocardial oxygen consumption (Chen, Traverse, Du, Hou, & Bache, 2002; Nordhaug et al., 2004; Suto et al., 1998). This is at least partly caused by vasoconstriction and subsequent disruption of the ventriculoarterial matching by elevated afterload without a concomitant increase in contractility (Nordhaug et al., 2004). The maximum stroke work at a given end‐diastolic volume will occur when Ees equals Eea (Figure 6). Stroke work efficiency (the fraction of total cardiac work, PVA, delivered to the circulation as stroke work), on the other hand, increases as the Ees/Ea ratio increases (Burkhoff & Sagawa, 1986). In these efficient states, a lower proportion of oxygen utilization is metabolized as potential energy (PE), where potential energy is the metabolism necessary to preserve the wall tension of the heart at end‐systole. This means that a higher proportion of metabolic oxygen consumption is used for energy transfer to the aorta, and the oxygen cost of stroke work is, therefore, lower (De Tombe, Jones, Burkhoff, Hunter, & Kass, 2017). The correlation between oxygen consumption and ventriculoarterial matching is well established (Hayashida et al., 1992; Nozawa, Yasumura, & Futaki, 1987), and also holds true in our study with a linear correlation between measured oxygen consumption and PVA.

In addition, NO has a distinct metabolic effect independent of loading conditions (Recchia et al., 1999). Various metabolic studies, as reviewed by Chang, Diers, and Hogg (2015), have demonstrated that the normal NO‐tone in tissues reduces the mitochondrial oxygen consumption by reducing the oxygen interaction with cytochrome c oxidase/complex I. However, there is only one study demonstrating a decrease in noncontractile oxygen utilization combined with an unchanged contractile efficiency (MVO_2_/PVA) after infusion of the NO‐precursor l‐arginine. In that study the authors also observed the predicted opposite effect by infusion of a NO synthetase inhibitor (Suto et al., 1998). By employing a large animal model (pigs) in our laboratory, we have not been able to reproduce a decrease in basal oxygen requirements in the post ischemic, stunned heart after infusion of l‐arginine, even though biochemical assessments demonstrated a substantial turnover of l‐arginine to l‐citrulline and thus a concomitant production of NO (Andersen, Igumnova, Kildal, & Myrmel, 2012). When studying experimental cardiogenic shock study in our lab, however, L‐NAME was shown to increase the basal oxygen requirements in the heart (Nordhaug et al., 2004). The impaired stroke work efficiency induced by L‐NAME was not mirrored in our present study, since there was no surplus MVO_2_ when L‐NAME was given after Riociguat. This is in contrast to the effect of L‐NAME given alone in other studies, as this compound has been shown to induce increased unloaded oxygen consumption (Nordhaug et al., 2004; Suto et al., 1998). Of notice, in this study we have not included a separate group of NO‐blockade alone (L‐NAME) due to the consistent energetic effect observed with this intervention.

Our study shows that Riociguat given to healthy animals has only a minor effect in the pulmonary circulation with no impact on the right ventriculo‐pulmonary arterial coupling. Riociguat (Adempas®) has been approved for treatment in pulmonary hypertension based on pulmonary vasodilatory effects in animal disease models and human studies. The effect of this sGC stimulator is therefore different in healthy and diseased lung vasculature.

## 5 CONCLUSION

Riociguat alone has no effect on myocardial oxygen consumption in the healthy heart. Both ventriculoarterial coupling and basal myocardial oxygen metabolism are unaffected by infusion of 100 µg/kg Riociguat. However, the subsequent administration of L‐NAME did not increase the relative oxygen consumption in the myocardium, and this suggests that the expected surplus basal MVO_2_ in the heart after blocking the endogenous NO‐tone by L‐NAME was attenuated by this dose of Riociguat. This oxygen‐conserving effect during L‐NAME stimulation indicates that Riociguat can induce “metabolic protection” in stressed myocardium and potentially also in pathological states. Such a potential should be explored in experimental disease models and during various pharmacological combinations.

## 6 DATA SHARING NOTICE

The data used for the study can be shared upon personal contact with the authors.

## CONFLICT OF INTEREST

None of the authors has any conflicts of interest.

## AUTHOR CONTRIBUTIONS

Torvind Næsheim: Drafting protocol, sourcing medications, setting up the lab, instrumentation, data collection, analysis, and authoring; Ole‐Jakob How: Idea, analysis, authoring; Truls Myrmel: Idea, drafting protocol, instrumentation, data collection, analysis, and authoring.

## ETHICAL STATEMENT

The research complies with the ethical guidelines of animal research as given by the the Norwegian Animal Research Authority. The protocol was approved in advance with the reference number FDF 2012/55972.
